# Recent Advances in Supported Metal Catalysts and Oxide Catalysts for the Reverse Water-Gas Shift Reaction

**DOI:** 10.3389/fchem.2020.00709

**Published:** 2020-08-31

**Authors:** Xiaodong Chen, Ya Chen, Chunyu Song, Peiyi Ji, Nannan Wang, Wenlong Wang, Lifeng Cui

**Affiliations:** ^1^School of Materials Science and Engineering, Dongguan University of Technology, Dongguan, China; ^2^Center for Clean Energy Technology, Faculty of Science, School of Mathematical and Physical Science, University of Technology Sydney, Sydney, NSW, Australia; ^3^Department of Applied Chemistry, School of Science, Xi'an Jiaotong University, Xi'an, China; ^4^School of Physical Science and Technology, ShanghaiTech University, Shanghai, China; ^5^College of Chemistry and Materials Science, Shanghai Normal University, Shanghai, China

**Keywords:** RWGSR, catalytic mechanism, catalytic performance, supported metal catalysts, oxide catalysts, chemical looping cycles

## Abstract

The reverse water-gas shift reaction (RWGSR), a crucial stage in the conversion of abundant CO_2_ into chemicals or hydrocarbon fuels, has attracted extensive attention as a renewable system to synthesize fuels by non-traditional routes. There have been persistent efforts to synthesize catalysts for industrial applications, with attention given to the catalytic activity, CO selectivity, and thermal stability. In this review, we describe the thermodynamics, kinetics, and atomic-level mechanisms of the RWGSR in relation to efficient RWGSR catalysts consisting of supported catalysts and oxide catalysts. In addition, we rationally classify, summarize, and analyze the effects of physicochemical properties, such as the morphologies, compositions, promoting abilities, and presence of strong metal-support interactions (SMSI), on the catalytic performance and CO selectivity in the RWGSR over supported catalysts. Regarding oxide catalysts (i.e., pure oxides, spinel, solid solution, and perovskite-type oxides), we emphasize the relationships among their surface structure, oxygen storage capacity (OSC), and catalytic performance in the RWGSR. Furthermore, the abilities of perovskite-type oxides to enhance the RWGSR with chemical looping cycles (RWGSR-CL) are systematically illustrated. These systematic introductions shed light on development of catalysts with high performance in RWGSR.

## Introduction

The increasing emissions of anthropogenic CO_2_ into our atmosphere through the unrestricted use of fossil fuels to drive industrial processes and human activity, particularly over the past few decades, has resulted in damage to the “carbon neutral” status of the earth and thus caused serious harm to the ecological system and to sustainable human development (Aresta et al., 2014). Therefore, the extensive efforts are needed to develop CO_2_ utilization technologies to address these issues (Mikkelsen et al., 2010). Benefiting from plentiful low-cost CO_2_ raw materials as well as the increasingly advanced CO_2_ capture and separation technologies, CO_2_ utilization is promising for commercial-scale applications (Aresta et al., 2016; Klankermayer et al., 2016).

The reverse water-gas shift reaction (RWGSR) is an indispensable part of CO_2_ utilization because it is a non-fossil route for providing feedstock for important chemical processes, such as methanol synthesis (Gao et al., 2016; Huš et al., 2017), Fischer-Tropsch synthesis (Riedel et al., 1999), and Monsanto/Cativa acetic acid synthesis (Maitlis et al., 1996; Jones, 2000). When it is used as an intermediate step in the direct thermochemical transformation of CO_2_ to hydrocarbons, such as methane (Sahebdelfar and Takht Ravanchi, 2015; Avanesian et al., 2016), ethanol (Sahebdelfar and Takht Ravanchi, 2015), low-carbon olefin (Liu et al., 2008; Zheng et al., 2017), and dimethyl ether (Centi and Perathoner, 2009), the RWGSR renders the process more practical. An important workable application of the RWGSR is associated with scarce H_2_ reutilization in the Mars Exploration Program, in which it could regenerate H_2_O more easily for astronauts to utilize (Avanesian et al., 2016). In biomass-based solid oxide fuel cells, the ratio of CO_2_/CO/H_2_ in the biomass gas can be considerably dictated by the RWGSR to realize its maximum energy storage efficiency (Chen et al., 2017a). Additionally, the RWGSR can be used to couple CO_2_ with alkylene oxide or low alkanes to generate valuable chemicals, including ethylene glycol (Arunajatesan et al., 2001), styrene (Burri et al., 2007; Batista et al., 2010), and light olefins (Mukherjee et al., 2016; Kang et al., 2017). In contrast to the direct thermal cracking process, these coupled reactions can effortlessly break the thermodynamic equilibrium constraints and effectively accelerate their utilization (Reddy et al., 2008; Rao et al., 2009).

The chemically inert CO_2_, with its high C-O bond energy of 806 kJ mol^−1^, enables the chemical transformation of CO_2_ to CO via the RWGSR (Wang et al., 2011). According to activation theory, the adsorption of CO_2_ on the oxygen vacancy sites of certain catalysts initiates the first step of the RWGSR when it involves the cleavage of its own C-O bond under thermal energy-driven conditions (Su et al., 2019). There are two idiographic activation mechanisms proposed for the production of CO from the RWGSR based on experimental observations and theoretical calculations (Goguet et al., 2016). The first pathway is CO_2_ hydrogenation to CO via the RWGSR, which proceeds via more reactive carboxyl (COOH^*^) or formate (HCOO^*^) intermediates, and the other pathway is the decomposition of CO_2_ to CO^*^ + O^*^via the direct C-O bond cleavage pathway (Weatherbee and Bartholomew, 1984; Kattel et al., 2016a). Once activated, these adsorbed intermediates will be instantaneously dissociated or desorbed on the constructed active centers of these catalysts to form the CO product (Tang et al., 2009; Roiaz et al., 2016). Based on this objective analysis, it is imperative to develop effective catalysts for CO_2_ activation in the RWGSR.

In this review, we concentrate on the catalytic performance of the RWGSR with two major categories of heterogeneous catalysts, including supported metal catalysts and oxide catalysts, which is a subject of increasing interest. Utilizing the thermodynamics and kinetics analyses and the atomic-level mechanisms, the principles of RWGSR catalyst design will be comprehensively described. In addition, the physicochemical properties of supported catalysts, such as the morphologies, compositions, promoting abilities, and presence of metal-support interactions, which affect the catalytic activity and CO selectivity of the RWGSR, will be systematically introduced to elucidate the structure-activity relationships. The relationships among the surface structure, oxygen storage capacity (OSC) and catalytic performance of oxide catalysts in the RWGSR are highlighted, especially for the application of perovskite-type oxides to enhance the RWGSR-CL. The present review provides general guidelines for the state-of-the-art architecture of heterogeneous catalysts for the RWGSR and a discussion of their challenges and further prospects.

## Thermodynamic Analysis

Since CO is arguably the most important C1-builiding block, the synthetic route of “CO_2_-to-CO” is considered an economical and valuable strategy (Barnard, 2008; Brennführer et al., 2009; Wu et al., 2011). Based on the thermodynamic standard enthalpy, the transformation of CO_2_ to CO via the RWGSR is more thermodynamically favorable at elevated temperature because it is reversible and endothermic and because its chemical equilibrium is pressure independent, as shown in Equation (1). However, the RWGSR is always accompanied by undesired CO_2_ methanation over the catalysts because of its excessive hydrogenation under ambient pressure (Kim et al., 2015; Ishito et al., 2016; Zhou et al., 2017). In addition, methanation is exothermic, favored at lower temperature, and pressure dependent, as shown in Equation (2).

(1) $$\begin{array}{l} \text{C}{\text{O}}_{2}+{\text{H}}_{2}\to \text{CO}+{\text{H}}_{2}\text{O }\Delta_{\text{r}}H^{\text{θ}}\left(298.15\text{K}\right)\\ \;=+\;40.6\text{ kJ mo}{\text{l}}^{-1}. \end{array}$$

(2) $$\begin{array}{l} \text{C}{\text{O}}_{2}+4{\text{H}}_{2}\to \text{C}{\text{H}}_{4}+2{\text{H}}_{2}\text{O }\Delta_{\text{r}}H^{\text{θ}}\left(298.15\text{K}\right)\\ \;=-165.0\text{ kJ mo}{\text{l}}^{-1}. \end{array}$$

**Figure 1:**
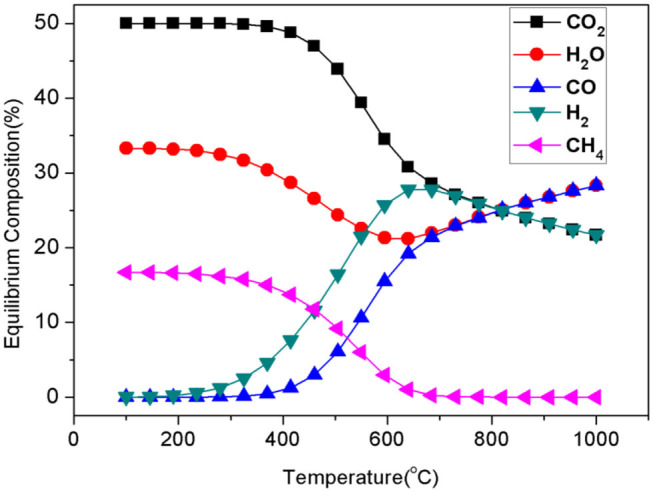
Equilibrium composition of the RWGSR and CO_2_ methanation (CO_2_:H_2_ = 1:1).

For both the parallel and the cascade reactions over the catalysts, the CO yield is seriously restricted to H_2_ utilization in additional competitive methanations. From the thermodynamic standpoint, as shown in Figure 1, the equilibrium composition favors the production of CH_4_ rather than that of CO in the RWGSR at lower temperatures. Therefore, it is challenging to construct heterogeneous catalysts to restrict the production of undesirable CH_4_ as a lower value-added by-product for applications at lower temperatures.

## Mechanism

The well-known catalytic mechanisms proposed for the RWGSR reaction can be classified into two categories: surface redox mechanisms and associative mechanisms (Su et al., 2017). The major difference between these mechanisms is whether the dissociated H_2_ species is involved in the formation of the carbon-containing intermediates, namely, formats, and carboxyls (Lin et al., 2017).

### Surface Redox Mechanism

The alternative oxido-reduction of active sites on the catalyst surface in the atmosphere of CO_2_/H_2_ feedstock is believed to be a prerequisite for the sustainability of the RWGSR. For Cu-based catalysts, e.g., the reaction mechanism can be described as follows (Chen et al., 2000; Xu and Ge, 2016):

(3) $$\begin{array}{l} \text{C}{\text{O}}_{2}\left(\text{g}\right)+2\text{C}{\text{u}}^{0}\left(\text{s}\right)\to \text{CO}\left(\text{g}\right)+\text{C}{\text{u}}_{2}\text{O}\left(\text{s}\right) \end{array}$$

(4) $$\begin{array}{l} {\text{H}}_{2}\left(\text{g}\right)+\text{C}{\text{u}}_{2}\text{O}\left(\text{s}\right)\to {\text{H}}_{2}\text{O}\left(\text{s}\right)+2\text{C}{\text{u}}^{0}\left(\text{s}\right). \end{array}$$

In these reactions, Cu^0^, the active site in the RWGSR, is involved in the rate-controlling step, CO_2_ reduction. The CO_2_ oxidizes the Cu^0^ to generate Cu^+^ and CO, while the H_2_ reduces the Cu^+^ to Cu^0^ to form H_2_O; thus, the reaction conforms to a redox mechanism. When the whole catalytic process of the RWGSR is considered in detail, the mechanism of the surface redox reaction can be decomposed into the following basic steps (“^*^” denotes the vacancy sites) (Gines et al., 1997; Fornero et al., 2017):

(5) $$\begin{array}{l} \text{C}{\text{O}}_{2}\left(\text{g}\right)+2^{*}\to \text{C}{\text{O}}^{*}+{\text{O}}^{*} \end{array}$$

(6) $$\begin{array}{l} \text{C}{\text{O}}^{*}\to \text{CO}\left(\text{g}\right){+}^{*} \end{array}$$

(7) $$\begin{array}{l} {\text{H}}_{2}\left(\text{g}\right)+2^{*}\to 2{\text{H}}^{*} \end{array}$$

(8) $$\begin{array}{l} 2{\text{H}}^{*}+{\text{O}}^{*}\to {\text{H}}_{2}{\text{O}}^{*}+2^{*} \end{array}$$

(9) $$\begin{array}{l} {\text{H}}_{2}{\text{O}}^{*}\to {\text{H}}_{2}\text{O}\left(\text{g}\right){+}^{*}. \end{array}$$

**Figure 2:**
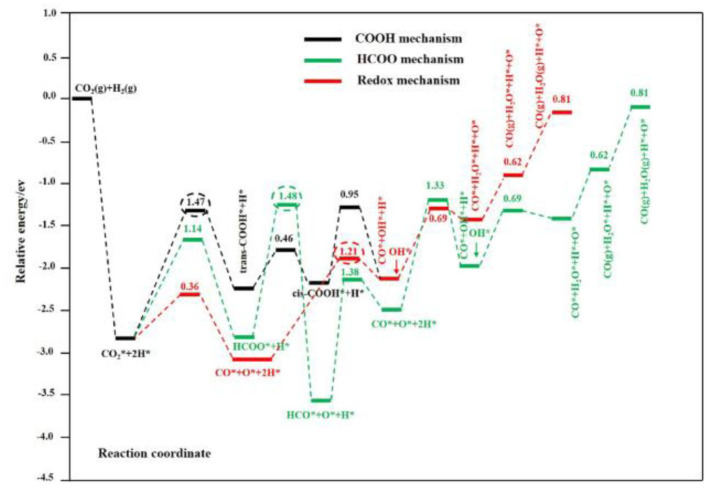
Calculated potential energy profile of the most favorable redox (red line), HCOO (green line), and COOH (black line) mechanisms for the RWGSR on the Cu@Mo2C (001) surface. The numbers in the figure are the activation barriers of the elementary steps. The numbers in red, green, and black circles are the activation barriers of the rate-limiting steps in the redox, HCOO, and COOH pathways, respectively. Reprinted with permission from Jing et al. (2019). Copyright (2019) American Chemical Society.

The study of kinetics is an important tool for establishing the redox mechanism of the RWGSR. Based on the Monte Carlo method to approximately simulate the RWGSR process over the Cu-based catalysts, CO_2_ dissociates immediately to give CO and adsorbed oxygen species and is then reduced by H_2_ with equivalent stoichiometric coefficients (Gines et al., 1997; Xu and Ge, 2016). In this process, the dissociative adsorption of CO_2_ on the Cu particles is the rate-determining step, and the reduction of the adsorbed oxygen-containing species and surface hydroxyls follows (Fujita et al., 1992; Wang et al., 2013b). Real-time temporal analysis of the products confirms that a surface-reduced Au/CeO_2_ catalyst can be reoxidized by exposure to CO_2_ pulses and that the surface oxygen deposited in this way can be reactively removed again, which is a prerequisite for the redox mechanism in the RWGSR. Furthermore, neglecting the changes in the hydroxyls and H_2_O on the surface imposed by the presence of H_2_ in the feed, the activity for active oxygen deposition is sufficient to make the redox mechanism the dominant reaction pathway (Fornero et al., 2017). Realistically, the RWGSR proceeds though a redox mechanism over Au/TiO_2_ catalysts in which the existing surface hydroxyls, surface Ti^3+^, and oxygen vacancies can jointly participate in the formation of a hydroxycarbonyl intermediate, which quickly decomposes to CO (Bobadilla et al., 2018). According to Density Functional Theory (DFT) calculations, the RWGSR on Cu@Mo_2_C (001) is preferentially selective for CO *via* a redox mechanism, and compared to the reaction *via* a COOH mechanism, the HCOO mechanism is kinetically less favorable due to its higher activation barrier in the rate-determining step, as shown in Figure 2. In the same way as the redox mechanism, the RWGSR occurs first by spontaneous dissociation of H_2_ to form H^*^, second by CO^*^ and O^*^ formation from the direct C-O bond cleavage of molecular CO_2_, third by the reaction of H^*^ and O^*^ to produce OH^*^, fourth by the reaction of two OH^*^ species to generate H_2_O^*^, and finally by the desorption of CO and H_2_O gas on the Cu@Mo_2_C (001) catalyst. Notably, the step for OH^*^ formation rather than CO^*^ formation in the redox mechanism, which has a higher activation barrier of 1.4 eV, is the rate-determining step (Jing et al., 2019).

### Associative Mechanism

#### Formate Species

The RWGSR pathway involves a formate (HCOO^*^) intermediate that is formed by the initial ${{\text{CO}}_{2}}^{*}$ hydrogenation step and subsequently undergoes an instantaneous dissociation reaction to produce CO (Arunajatesan et al., 2007; Cao et al., 2016; Chen et al., 2016; Wolf et al., 2016). The reaction is described by the following steps (“^*^” denotes the vacancy sites) (Chen et al., 2017a,b):

(10) $$\begin{array}{l} {\text{H}}_{2}\left(\text{g}\right)+2^{*}\to {\text{H}}^{*}+{\text{H}}^{*} \end{array}$$

(11) $$\begin{array}{l} \text{C}{\text{O}}_{2}\left(\text{g}\right){+}^{*}\to {{\text{CO}}_{2}}^{*} \end{array}$$

(12) $$\begin{array}{l} {{\text{CO}}_{2}}^{*}+{\text{H}}^{*}\to \text{HCO}{\text{O}}^{*}{+}^{*} \end{array}$$

(13) $$\begin{array}{l} \text{HCO}{\text{O}}^{*}\left(\text{COO}{\text{H}}^{*}\right){+}^{*}\to \text{HC}{\text{O}}^{*}\left(\text{CO}{\text{H}}^{*}\right)+{\text{O}}^{*} \end{array}$$

(14) $$\begin{array}{l} \text{HC}{\text{O}}^{*}\left(\text{CO}{\text{H}}^{*}\right){+}^{*}\to \text{C}{\text{O}}^{*}+{\text{H}}^{*} \end{array}$$

(15) $$\begin{array}{l} {\text{H}}^{*}+{\text{O}}^{*}\to \text{O}{\text{H}}^{*}{+}^{*} \end{array}$$

(16) $$\begin{array}{l} {\text{H}}^{*}+\text{O}{\text{H}}^{*}\to {\text{H}}_{2}{\text{O}}^{*} \end{array}$$

(17) $$\begin{array}{l} {\text{H}}_{2}{\text{O}}^{*}\to {\text{H}}_{2}\text{O}\left(\text{g}\right){+}^{*} \end{array}$$

(18) $$\begin{array}{l} \text{C}{\text{O}}^{*}\to \text{CO}\left(\text{g}\right){+}^{*}. \end{array}$$

As indicated by the temperature-programmed desorption spectra of H_2_/CO_2_ co-adsorbed on Cu/SiO_2_ and Cu/K/SiO_2_ catalysts, the H atoms either associate with CO_2_-Cu to form formates or migrate to the surfaces of the interfacial sites, resulting in the formation of K_2_O and CO by decomposition (Chen and Cheng, 2002; Chen et al., 2003). Regarding the chemical state of Cu in the RWGSR, the Cu^0^ and Cu^+^ atoms are proposed to coexist on the Cu-based catalyst surface, and their roles are possibly to dissociate H_2_ and stabilize the formats, respectively (Chen et al., 2000). Based on transient diffused reflection Fourier transform infrared spectroscopy (DRIFTS), the appearance of interfacial sites may result from an electron transfer from the Pt to the neighboring O in the KO_x_ species over the Pt/K/mutille and Pt/K/L catalysts, which are responsible for the decomposition of the formates to produce CO (Liang et al., 2017; Yang et al., 2017). The *in-situ* DRIFTS also shows that when strong basic sites, such as those of KOH, are introduced into Ni/Al_2_O_3_ catalysts, the formates instead of the carbonates are strongly absorbed on their surface, promoting the hydrogenation of CO_2_ to CO *via* the RWGSR (Zhang et al., 2019a). For Cu/CeO_2_-nanorode catalysts, *in-situ* DRIFTS points to bidentate formate as the active intermediates for the RWGSR because the preferential formation of a high bidentate formate coverage on their surface may have the surface geometry of a CeO_2_ (110) termination in which the nearest surface oxygen distance is 2.71Å, which is a more suitable spacing for the formation of bidentate formates and could thus be the main reason for the excellent performance (Lin et al., 2018a,b). The results from *in-situ* DRIFTS indicate that the CO_2_ must first react with the surface hydroxyls on Al_2_O_3_ to form bicarbonates, which subsequently react with the adsorbed H on Ru or Au to produce adsorbed formates, most likely at the metal/oxide interface, and then react rapidly with the adsorbed H to form CO (Wang et al., 2016a; Bobadilla et al., 2018). Based on a steady-state isotopic transient kinetic analysis, the disappearance trend of the infrared signatures of H^12^COO^*^ is consistent with the MS signals of ^12^CO products when the feed gas is switched from ^12^CO_2_/H_2_/Ar to ^13^CO_2_/H_2_/Ar over a Pd/Al_2_O_3_ catalyst, suggesting that H^12^COO^*^ is the reactive intermediate rather than a spectator, and the rate-determining step for the CO formation is related to HCOO^*^ (Wang et al., 2017). DFT calculations demonstrate that a formate mechanism is feasible for the RWGSR catalyzed by M_1_/W_6_S_8_ (M = Fe, Ru, and Os). More concretely, HCOO^*^ formation starts with the most stable adsorption configuration of ${{\text{CO}}_{2}}^{*}$, in which an H^*^ is placed on the S site, and H^*^ tends to attack the C atom of CO_2_ to form a C-H bond. When HCOO^*^ is formed, it is hydrogenated to form a HCOOH^*^ intermediate with a relatively low activation energy barrier, and the conversion will proceed to form the final product, CO. In the overall process, the H_2_ dissociation on M_1_/W_6_S_8_ (M = Fe, Ru, and Os) is the rate-determining step (Zhang et al., 2018).

#### Carboxyl Species

The carbonyl species, the predominately active intermediate, is selectivity produced through activation of the C-O bond of the CO_2_ molecule followed by H-assisted formation of COOH^*^ (Tibiletti et al., 2004; Kim et al., 2012a,b). This intermediate immediately decomposes into CO by two different pathways, as shown below (“^*^” denotes the vacancy sites) (Chen et al., 2017a,b).

**Table:** 

(I) H_2_(g)+2^*^ → H^*^+H^*^	(1)	(II) H_2_(g)+2^*^ → H^*^+H^*^	(1)
CO_2_(g)+^*^ → ${{\text{CO}}_{2}}^{*}$	(2)	CO_2_(g)+^*^ → ${\text{CO}}_{2^{*}}$	(2)
${{\text{CO}}_{2}}^{*}+$H^*^ → (COOH^*^)+^*^	(3)	${{\text{CO}}_{2}}^{*}+$H^*^ → (COOH^*^)+^*^	(3)
COOH^*^+^*^ → CO^*^+OH^*^	(4)	COOH^*^+^*^ → HCO^*^(COH^*^)+O^*^	(4)
H^*^+OH^*^ → H_2_O^*^	(5)	HCO^*^(COH^*^)+^*^ → CO^*^+H^*^	(5)
H_2_O^*^ → H_2_O(g)+^*^	(6)	H^*^+O^*^ → OH^*^+^*^	(6)
CO^*^ → CO(g)+^*^	(7)	H^*^+OH^*^ → H_2_O^*^	(7)
		H_2_O^*^ → H_2_O(g)+^*^	(8)
		CO^*^ → CO(g)+^*^	(9)

**Figure 3:**
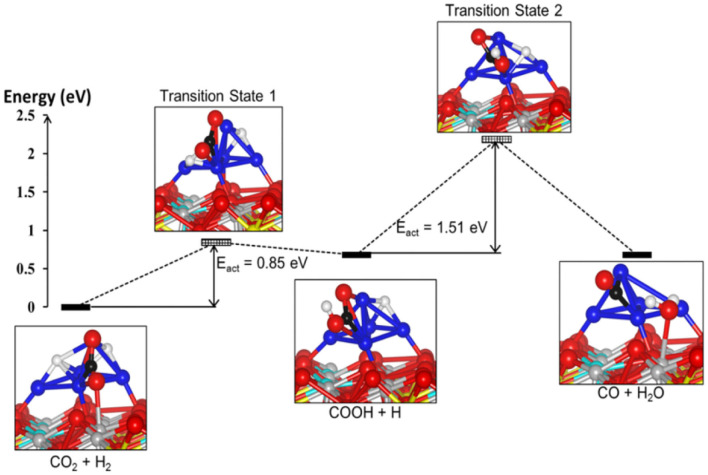
Energy profile showing the reactants, intermediates, transition states, and final products for the RWGSR on the Ni_5_/YSZ(111) interface (Color key: O, Zr, Y, Ni, C, and H are represented by red, gray, cyan, blue, black, and white spheres, respectively. The yellow spheres represent the oxygen vacancies). Reprinted with permission from Cadi-Essadek et al. (2018). Copyright (2018) American Chemical Society.

On the basis of *in-situ* Fourier transform infrared spectroscopy (FT-IR) analyses, a Pt/TiO_2_ catalyst treated at a high temperature and possessing reducible TiO_2_ sites but no Pt sites is exclusively active for CO product, and thus the carboxyl species formed on the reducible TiO_2_ sites are the intermediates in the formation of CO in the RWGSR (Kim et al., 2013). H/D isotopic substitution and kinetics and the results of the *in-situ* DRIFTS experiments illustrate that the CO formation proceeds *via* a mechanism in which H assists the dissociation of the C-O bond and that a carboxyl is a more plausible intermediate than is a formate. In addition, the formates is still present on the Cu surface under the reaction conditions, but a fraction of them can be considered spectators of the reaction mechanism (Karelovic et al., 2019). The results from transient DRIFT-MS steady-state isotopic transient kinetic analysis analyses indicate that the characteristic exchange time (defined here as the time at which the DRIFTS signal of the intermediate decreases by 50% following the isotopic switch) of the carboxyl species agrees with that of the CO product (defined here as the time needed to achieve 50% exchange between the two isotopes, e.g., ^12^CO(g) and ^13^CO(g) of the main reaction product from MS measurements) when the feed gas is switched from ^13^CO_2_/H_2_ to ^12^CO_2_/H_2_ over the Pt/CeO_2_ catalyst. These data quantitatively demonstrated that, for the present catalyst and conditions, the main reaction pathway is the formation of CO from the carboxyl species at the oxygen vacancies over the Pt-CeO_x_ interface (Goguet et al., 2004a). DFT calculations indicate that the formation of COOH^*^ over Mo_6_S_8_-TM (TM = Pd, Pt, Ag) nanoclusters by the binding of the H^*^ atom to the O atom of ${\text{CO}}_{2^{*}}$ followed by its decomposition to CO is very favorable. Note that the COOH^*^ dissociation over Mo_6_S_8_-Ag is the rate-determining step in the overall process, whereas the rate-determining step of Mo_6_S_8_-Pd and Mo_6_S_8_-Pt in the carboxyl pathway is the transition step of the H_2_ dissociation (Zheng et al., 2017). Moreover, DFT calculations show that the RWGSR complies with a carboxyl mechanism over a Ni_5_/YSZ (111) catalyst through the identification of the structures and calculation of the energies of the intermediate state and two transition states, as shown in Figure 3. It has been suggested that one of the H^*^ atoms migrates toward the nearest O atom of the ${{\text{CO}}_{2}}^{*}$ to form the COOH^*^ intermediate and subsequently involves its protonation, allowing the formation of H_2_O^*^ adsorbed on the surface and the CO^*^ adsorbed on the Ni cluster. This calculation also shows that the second transition state for the dissociation of the COOH^*^ intermediate is the rate-determining step of the overall pathway and has an energy barrier of 1.51 eV (Cadi-Essadek et al., 2018).

## Catalytic System

In recent decades, heterogeneous catalysts that promote the RWGSR have been extensively studied because of the gradual realization of their widespread application prospects for CO_2_ utilization. At the early stage, much research has focused on the oxide catalysts due to their effluent oxygen vacancies sites, such as CeO_2_, CuO, ZnO, Al_2_O_3_, Fe_2_O_3_, Cr_2_O_3_, In_2_O_3_, and MnO_2_ (Saeidi et al., 2017; Su et al., 2017; He et al., 2019). Although the CO selectivities of these oxide catalysts are desirable in RWGSR, their disadvantages of lower CO_2_ activation and feasible poisons and sintering are hindering their extended application. In order to address these issues, persistent studies have concentrated on the fabrication of composite oxides (i.e., CuO/ZnO/Al_2_O_3_, NiO/CeO_2_, ZnO/Al_2_O_3_, ZnO/Cr_2_O_3_, CuOx/CeO_2_, CuO–CeO_2_/SBA-15, In_2_O_3_-CeO_2_, FeO_x_, etc.) (Liu et al., 2015; Dai et al., 2018; Ronda-Lloret et al., 2018; Panarities et al., 2020), spinel oxides (i.e., ZnAl_2_O_4_, ZnCr_2_O_4_, CuAl_2_O_4_, CoAl_2_O_4_, etc.) (Joo and Jung, 2003; Bahmanpour et al., 2019, 2020), solid solution oxides (i.e., Zn_x_Zr_1−x_O_2−y_, Ce_0.5_Zr_0.5_O_2_, Ni_x_Ce_0.75_Zr_0.25−x_O_2_, etc.) (Zonetti et al., 2014), and perovskite-type oxides (i.e., BaZr_0.8_Y_0.16_Zn_0.04_O_3_, La_0.75_Sr_0.25_CoO_3−δ_, La_0.75_Sr_0.25_FeO_3_, La_0.75_Sr_0.25_Fe_1−Y_Cu_Y_O_3_, LaNiO_3_, La_0.9_Sr_0.1_NiO_3+δ_, La_0.9_Sr_0.1_FeO_3−δ_, La_0.9_Sr_0.1_Ni_0.5_Fe_0.5_O_3−δ_, La_0.75_Sr_0.25_Cr_0.5_Mn_0.5_O_3−δ_, SrCe_0.9_Y_0.1_O_3−δ_, etc.) (Yamazoe et al., 1982; Ten Elshof et al., 1996; Klvana et al., 1999; Yang and Lin, 2006; Radovic et al., 2008; Zhuang et al., 2019; Bogolowski et al., 2020; Liu et al., 2020), which have the approvable characteristics of both stable structure and reverse oxygen storage capacity to increase RWGSR performance.

Recently, considerable efforts have been devoted to the design of metal-based catalysts (i.e., Pt, Pd, Au, Rh, Ru, Cu, Ni, Re, Co, Fe, Mo, etc.) immobilized onto the metal oxide support material (i.e., CeO_2_, TiO_2_, Al_2_O_3_, ZnO, ZrO_2_, SiO_2_, etc.) (Goguet et al., 2004b; Wang et al., 2011; Liu et al., 2015; Álvarez Galván et al., 2016; Ro et al., 2016; Nielsen et al., 2018) because they have metal/oxide interfaces with high reducibility to facilitate CO_2_ activation in RWGSR. However, the CH_4_ may be easily produced on these catalysts in the case of excessive hydrogenation of the C-O bond of the CO_2_ molecule, which can be detrimental to CO selectivity (Yeung et al., 2005; Sun et al., 2015; Wang et al., 2016b; Kattel et al., 2017). In reality, an effective supported metal catalyst must be capable of both the C-O bond scission of CO_2_ and the appropriate hydrogenation. Thus, the supported metal catalysts must not only dissociate hydrogen relatively easily but also allow it to migrate onto the adjacent oxygen vacancies, where the adsorbed CO_2_ is further hydrogenated (Chen and Cheng, 2002; Wang et al., 2017). Furthermore, the details of the activity and selectivity of some representative RWGSR catalysts under reaction conditions are presented in Table 1.

**Table 1:** Summary of the reaction conditions with conversion to and selectivity for CO, when available, for selected RWGSR catalysts.

**Catalyst**	**Catalyst mass (mg)**	**H_**2**_/CO_**2**_ ratio**	**WHSV (mL g_cat^−1^_ h^−1^)**	**Temperature (°C)**	**Pressure (MPa)**	**Conversion (%)**	**Selectivity (%)**
2%Pt/CeO_2_ (Goguet et al., 2004b)	40	4:1	300,000	290	0.1	21.7	~100
1%Ni/CeO_2_ (Wang et al., 2013a)	50	1:1	120,000	400	0.1	~4.5	~90
5%Ru/CeO_2_ (Panaritis et al., 2018)	50	1:1	120,000	350	0.1	~16	~31
RuNi/CeZr (Sache et al., 2020)	250	4:1	24,000	350	0.1	53	93
FeNi/CeZr (Sache et al., 2020)	250	4:1	24,000	350	0.1	13	60
5%Ru/Sm-CeO_2_ (Panaritis et al., 2018)	50	1:1	120,000	350	0.1	~16	~69
3.2%PtCo/CeO_2_ (Kattel et al., 2016b)	N/A	2:1	N/A	300	0.1	9.1	92.3
PdNi/CeO_2_ (Porosoff et al., 2014)	100	7:1	30,000	300	1.07 × 10^−4^	2.5	37.5
10%Co/CeO_2_ (Wang et al., 2013b)	20	3:1	300,000	300	0.1	3.8	39.4
1%Pt/TiO_2_ (Kim et al., 2012b)	500	1:1	12,000	300	0.1	~13	~100
3.2%PtCo/TiO_2_ (Kattel et al., 2016b)	N/A	2:1	N/A	300	0.1	8.2	98.8
0.2%Rh/TiO_2_ (Matsubu et al., 2015)	15	4:1	40,000	200	0.1	N/A	~14.5
0.1%Ru/Al_2_O_3_ (Matsubu et al., 2015)	50	3:1	720,000	400	0.1	~13	~80
1%Pt/Al_2_O_3_ (Kim et al., 2012b)	500	1.43:1	12,000	300	0.1	~5.8	~100
Ni-Mo/Al_2_O_3_ (Kharaji et al., 2014)	N/A	1:1	30,000	600	0.1	~35	N/A
Mo/Al_2_O_3_ (Kharaji et al., 2014)	N/A	1:1	30,000	600	0.1	~15	N/A
Fe-Mo/γ-Al_2_O_3_ (Kharaji et al., 2013)	5,000	1:1	N/A	400	0.1	~22	~100
Fe/γ-Al_2_O_3_ (Kharaji et al., 2013)	5,000	1:1	N/A	400	0.1	~15.5	~100
PtCo/γ-Al_2_O_3_ (Porosoff and Chen, 2013)	N/A	3:1	N/A	300	4 × 10^−3^	10	89.4
20%Cu-Ni/γ-Al_2_O_3_ (Liu and Liu, 1999)	N/A	1:1	2,000	500	0.1	23.2	75.5
3.2%PtCo/ZrO_2_ (Kattel et al., 2016b)	N/A	2:1	N/A	300	0.1	7.8	89.5
0.5%Pd/La_2_O_3_/MWCNT (Kwak et al., 2013a)	50	3:1	72,000	400	0.1	~20	~100
0.5%Pd/MWCNT (Kwak et al., 2013a)	50	3:1	72,000	400	0.1	0	0
10%Cu-0.3%Fe/SiO_2_ (Chen et al., 2004)	20	1:1	120,000	600	0.1	~12	~100
10%Cu/SiO_2_ (Chen et al., 2004)	20	1:1	120,000	600	0.1	~8	~100
0.3%Fe/SiO_2_ (Chen et al., 2004)	20	1:1	120,000	600	0.1	~2	~100
1%NiO/CeO_2_/SBA-15 (Lu and Kawamoto, 2014)	2,000	1:1	1,500	450	0.1	~2.5	100
ZnO/Al_2_O_3_ (Zn:Al = 1:2) (Park et al., 2001)	N/A	3:1	15,000	400	0.1	~3.4	~100
2D (δ)-MnO_2_ (He et al., 2019)	30	1:1	40,000	850	0.1	50	100
ZnO (Park et al., 2001)	N/A	3:1	15,000	400	0.1	~2.6	~100
Al_2_O_3_ (Park et al., 2001)	N/A	3:1	15,000	400	0.1	0	100
1%Cu/β-Mo_2_C (Zhang et al., 2016)	20	2:1	300,000	350	0.1	11	40
7.5%Co-Mo_2_C (Porosoff et al., 2014)	100	2:1	36,000	300	1.07 × 10^−4^	9.5	~98.1
Mo_2_C (Porosoff et al., 2014)	100	2:1	36,000	300	1.07 × 10^−4^	8.7	~93.5

### Supported Metal Catalysts

The supported catalysts that can be seriously considered in the RWGSR due to their bifunctional catalytic roles for CO_2_ activation and appropriate hydrogenation (Porosoff et al., 2016). However, RWGSR is demonstrated to be structure sensitive reaction; thus, the CO selectivity of which can be dictated by tailoring the structure functionality of supported catalysts through the SMSI effect, metal size effect, shape and crystal face effect, bimetallic effect, and alkali promoter effect to boost their concentrated activity.

#### Strong Metal-Support Interaction (SMSI) Effect

The importance of support has been increasingly recognized in the decades following the discovery of SMSIs (Garin, 2001; Diebold, 2003; Neophytides et al., 2005; Fu and Wagner, 2007; Liu et al., 2013). In addition to dispersing metallic particles, the support also functions to influence the catalytic properties of the supported metal catalysts through geometric or electronic effects (Naito et al., 2006; Krstajić et al., 2008; Delgado et al., 2011; Li et al., 2015). In this section, the mechanisms by which SMSI effects provide catalytic characteristics of supported catalysts for the RWGSR are further elucidated.

**Figure 4:**
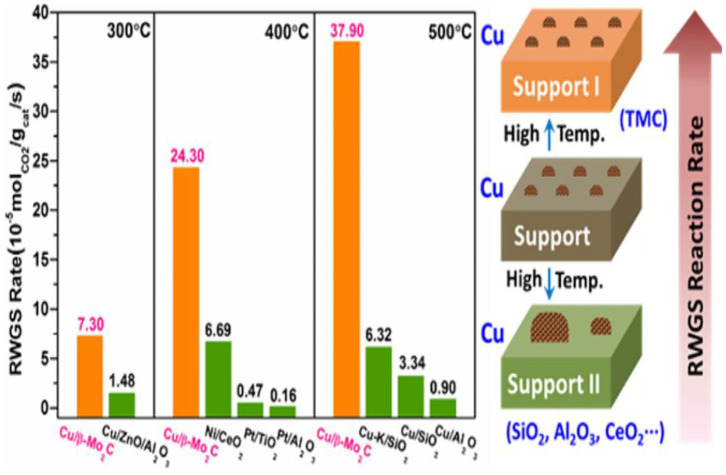
RWGSR rate and SMSI effect of Cu/β-Mo_2_C and reference catalysts. Reprinted with permission from Zhang et al. (2016). Copyright (2017) American Chemical Society.

The high electron donating property of metallic Pt in contact with a Ti^3+^ ion site is caused by the SMSI effect, which generates new Pt-O_v_-Ti^3+^ sites for CO production over the Pt/TiO_2_ catalyst (Kim et al., 2012a,b, 2013). Additionally, by replacing the ZrO_2_ support by TiO_2_, the SMSI effect selectively weakens the binding of the C-O bond and O-bond intermediates at the PtCo-oxide interface, thus leading to the high selectivity toward CO in the RWGSR (Kattel et al., 2016b). For TiO_2_-supported Rh catalysts, an adsorbate-mediated SMSI (A-SMSI) encapsulation state can be formed as a result of its treatment in a 20CO_2_:2H_2_ environment at 250°C. The high coverage of the adsorbates (HCO_x_) on the support induces oxygen vacancy formation, driving the migration of the HCO_x_-functionalized support onto the metal. This A-SMSI encapsulation state is more stable against reoxidation by H_2_O in the RWGSR process compared with the SMSI encapsulation state formed as a result of only H_2_ treatment, which modifies the reactivity of all the remaining exposed Rh sites and appears to be comprehensive in covering the Rh but permeable to reactants, due to its amorphous properties. Consequently, formation of the A-SMSI state induces a selectivity switch in the CO_2_-reduction reaction from the CH_4_ production on the bare Rh particles to the CO product in the A-SMSI state, thus effectively rendering Rh less active for C-H bond formation (Matsubu et al., 2017). For Ir/CeO_2_ catalysts, the SMSIs can enable more oxygen atoms to be incorporated into the metal surface, resulting in a weaker CO adsorption strength over the partially oxidized Ir nanoparticles and giving a near 100% selectivity toward CO compared with that over the corresponding metallic Ir. Therefore, modulation of the chemical state of the metal species by the SMSI is more important for the regulation of the observed CO selectivity in the RWGSR (Li et al., 2017). For the Cu/CeO_2_ catalyst, the Ce^3+^-O_v_-Cu^0^ and Cu^0^-CeO_2−δ_ interface structures can be generated by the electron transfer from Cu to Ce on its surface through SMSI effect, which can boost the adsorption and activation performance of reactant CO_2_ and H_2_ molecules for RWGSR (Zhou et al., 2020). In the simulation of catalytic CO_2_ reduction by Pd-decorated silicon-hydride nanosheets (Pd@SiNS), the direct SMSI between the Pd nanoparticle and the Si nanosheet causes H transfer from the Pd to the oxidized SiNS surface, which may occur repeatedly by two mechanisms. First, an H atom adsorbed on the Pd nanoparticle interacts with a surface Si-O-Si and creates a Si-OH; second, another H from the Pd nanoparticle forms a bond with the Si-OH, which leads to desorption of the H_2_O, creating a surface radical, thereby enabling a catalytic cycle. Furthermore, the strain induced in the SiNS by the Si-O-Si bonds enhances the reactivity of the oxidized SiNS surface toward the transformation of CO_2_ to CO under mild conditions (Qian et al., 2019). In the context of Mo_2_C-supported Co catalysts, the SMSI effect facilitates the formation of the amorphous CoMoC_y_O_z_ phase formed during the CO_2_ hydrogenation, in which the Co with a positive charge is identified as the critical active site that dissociates CH_4_ to CO. Therefore, the addition of 7.5% Co to Mo_2_C leads to an increase in conversion from 8.7 to 9.5%, while the CO:CH_4_ ratio increases from 15 to 51 (Porosoff et al., 2014). When Cu is added to the β-Mo_2_C support during the preparation process, the SMSI effect not only promotes the dispersion of supported copper and prevents the aggregation of Cu particles but also enables a portion of the electrons to transfer from Cu to Mo_2_C so that the Cu^+^ and Cu^0^ species coexist in the Cu/β-Mo_2_C catalyst. Its modulated electronic structure makes the highly dispersed Cu species more active in the CO_2_ activation and accelerates the CO^*^ desorption in the following transformation reactions, which accounts for its excellent activity in the RWGSR, as depicted in Figure 4 (Zhang et al., 2016).

#### Metal Size Effect

The RWGSR is considered structure sensitive for supported catalysts, of which the intermediate dissociation pathway associated with H assistance is substantially dependent on the size of the anchored metal active sties and thus exerts an influence on the CO selectivity of the RWGSR (Chen et al., 2017a).

**Figure 5:**
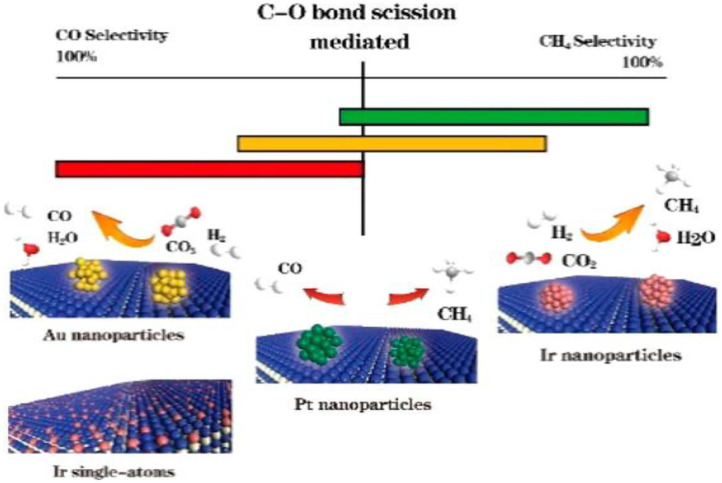
Comparative CO and CH_4_ selectivities of the Ir_1_/TiO_2_, Ir_5_/TiO_2_, Pt_5_/TiO_2_, and Au_5_/TiO_2_ catalysts. Reprinted with permission from Chen et al. (2017a). Copyright (2017) American Chemical Society.

Multiple studies have been conducted to study the size effect of metal sites relative to CO selectivity in supported noble metal catalysts. The metal active sites (i.e., Pt, Pd, Ru) dispersed at an atomic level contribute more to the CO product compared to metal clusters at a 3D level. This phenomenon is a consequence of the absence of larger metals clusters in which the initially formed CO_ad_ can be further activated during the continuous reaction (Kwak et al., 2013a,b; Wang et al., 2016a; Chen et al., 2017a). In addition, the Pd sites that slightly retain the CO surface species formed from the formates and other intermediates are more prevalent on the surface of the smaller Pd particles and thus exhibit a higher selectivity toward the CO product. In contrast, the larger Pd particles, due to a higher population of terrace sites in which it is easier to form multi-bound CO and dissociated H_2_ bound in the vicinity of CO, reveal a stronger interaction with CO. These stable CO species are mainly in multi-bound forms and act as the direct intermediates to CH_4_ (Wang et al., 2015, 2017). Matsubu et al. have utilized DRIFTS with known site-specific extinction coefficients to quantify the fraction of Rh sites residing as atomically dispersed isolated sites (Rh_iso_), as well as Rh sites on the surface of Rh nanoparticles (Rh_NP_) for a series of TiO_2_ supported Rh catalysts. The reaction condition-induced disintegration of Rh_NP_, which form the Rh_iso_ active sites, have been observed to control the CO selectivity of the RWGSR (Matsubu et al., 2015). Furthermore, we have determined that the difference between the desorption energy and dissociation barrier of metal carbonyls is a critical factor for determining the CO selectivity of the RWGSR by combining DFT calculations and experiments, as shown in Figure 5. Specifically, narrowing the size of the Ir active sites by decreasing the Ir loading over Ir/TiO_2_ catalysts can hinder the carbonyl dissociation but improve the CO desorption, giving rise to CO selectivity (Chen et al., 2017a).

For supported non-noble metal catalysts, significant efforts on Ni-based catalysts have also shown that smaller anchored Ni active sites are beneficial to produce CO in the RWGSR (Wang et al., 2013a,d; Lu and Kawamoto, 2014). The consecutive pathway is favored on small Ni particles, which is attributed to low H_2_ coverage on the Ni surface, thus leading to the dissociation of the intermediates and high CO selectivity. Whereas the RWGSR on large Ni particles may be controlled by mixed consecutive and parallel pathways, it increases the likelihood that the intermediates will be competitively hydrogenated to CO or CH_4_ as part of a parallel reaction pathway (Wu et al., 2015). Millet et al. reported on the activation of CO_2_ on Ni single-atom catalysts that are synthesized using a solid solution approach by controlled substitution of 1–10 atom % of Mg^2+^ by Ni^2+^ inside the MgO structure. The Ni atoms are preferentially located on the surface of the MgO and, as predicted by hybrid-functional calculations, favor the low-coordinated sites, where they can reduce the strength of the CO_2_ binding and promote H_2_ dissociation. Thus, the Ni atoms are active for CO_2_ conversion through the RWGSR but are unable to conduct its further hydrogenation to CH_4_, for which Ni clusters are needed (Millet et al., 2019).

#### Shape and Crystal Face Effect

RWGSR activities are also significantly depending on the shape and exposed crystal face of catalysts because they can determine the virtual adsorption energy and desorption energy of intermediates in the reaction process (Liu et al., 2019).

Up to now, abundant efforts have been dedicated to study the effect of surface structure of Cu-based catalysts on RWGSR performance. Through the simulation of the adsorption of CO_2_, H_2_, H, O, OH, CO, and H_2_O on the Cu(*hkl*) surfaces at low coverage, it has been demonstrated that the trend in the calculated activation barriers for the reaction is CO_2_ dissociative adsorption (namely CO_2, g_ COs + Os) follows the order of Cu(110)<Cu(100)<Cu(111), suggesting that the most efficient crystal surface for catalyzing RWGSR by copper is Cu(110), and the more densely packed Cu(111) surface is the least active among the Cu(*hkl*) surfaces studied here (Nakamura et al., 1998; Wang et al., 2003; Wang and Nakamura, 2010). When the Cu particles are doped onto the CeO_2_-Nanaorod and CeO_2_-Nanosphere surfaces, respectively, which can be marked as Cu/CeO_2_-NR(111) and Cu/CeO_2_-NS(110), by comparation, the Cu/CeO_2_-NR displays the higher RWGSR activity. This is mainly because that the CO_2_ dissociative activation and the formation of active bidentate carbonate and formate intermediates over CeO_2_(110) become more feasible (Kovacevic et al., 2016; Lin et al., 2018b). Furthermore, self-assembled CeO_2_ with 3D hollow nanosphere, nanoparticle, and nanocube morphologies are used to support Cu particles, which can be denoted as Cu/CeO_2_-hs(111), Cu/CeO_2_-np(111), and Cu/CeO_2_-nc(200), respectively. Thereinto, the Cu/CeO_2_-hs(111) presents the best catalytic RWGSR performance among these as-prepared catalysts due to its high concentration of active oxygen vacancies sites (Zhang et al., 2020). For PtCo/TiO_2_(110), ^*^HCOO is formed as an intermediate, which may eventually produce CO, whereas for PtCo/CeO_2_(110), the aside from the route that proceeds *via* ^*^HCOO, a pathway *via* a ^*^CH_3_O intermediate is operating in parallel, which likely leads to the formation of CH_4_. Moreover, DFT calculation demonstrates that the adsorption of CO_2_ is stronger at the Ni_n_/YSZ(111) (*n* = 4–7, 10, and 20) interface than on the clean YSZ(111) between the Ni clusters and the YSZ(111) surface, which facilitates the transformation of CO_2_ to CO (Cadi-Essadek et al., 2018). For Cu@Mo_2_C(001) and Cu_4_@Mo_2_C(001) surfaces, although the dissociative adsorption of H_2_ on these two surface is barrier-free and highly exothermic, the activation barrier of carboxyl formation or C-O bond scission as a rate-limiting step on the Cu_4_@Mo_2_C(001) surfaces is smaller, and the desorption of CO at the Cu site needs less heat than Mo site, thereby accelerating CO_2_ conversion in RWGSR (Chen and Cheng, 2002).

#### Bimetallic Effect

The behavior of a catalyst is modulated by its interaction with other catalyst components, such as a second metal, which influences it through electronic interactions, generates interfacial active sites, or is directly involved in the reaction by bonding to reactants or intermediates (Liu and Liu, 1999; Liu et al., 2015). Therefore, supported bimetallic catalysts have been extensively used for the RWGSR due to their tuning catalytic activity that may be achieved by two metals working synergistically.

**Figure 6:**
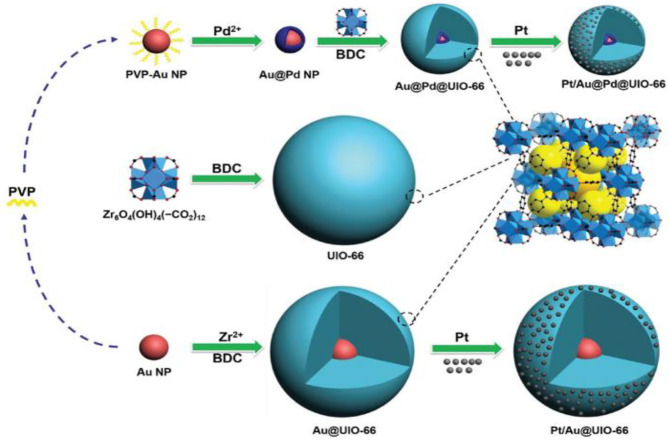
Synthetic route for the production of Au@Pd NPs and other nanocomposites. Reprinted with permission from Zheng et al. (2018). Copyright (2017) Wiley-VCH.

**Figure 7:**
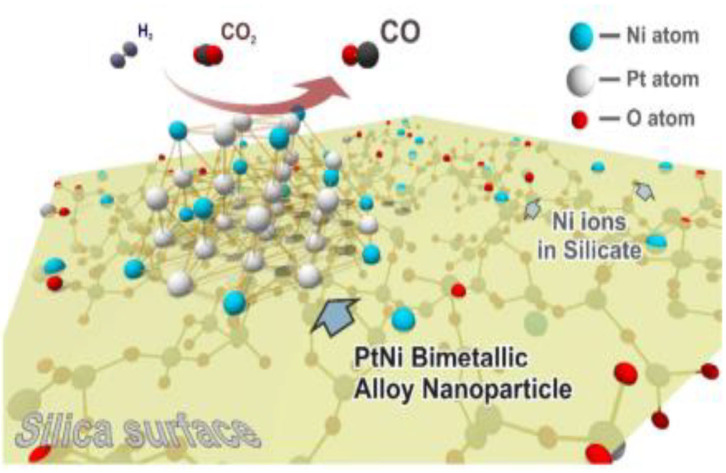
Schematic model of the PtNi/SBA-15 catalyst. Reprinted with permission from Liu et al. (2018). Copyright (2018) American Chemical Society.

The existence of Mo in the structure of the Fe-Mo/Al_2_O_3_ catalyst enhances its catalytic performance for the RWGSR due to the electronic effect, which transfers electrons from Fe to Mo and leads to an electron-deficient state of the Fe species, in which it is not helpful for CO adsorption and hence inhibits the continuous hydrogenation of the intermediates (Kharaji et al., 2013; Panaritis et al., 2018). It is reasonable for the Ni species with the electron deficient state to possess high catalytic performance for RWGSR when Ni is added as a second metal component to the Mo/Al_2_O_3_ catalyst (Kharaji et al., 2014). As for RuNi/CeZr catalyst, the addition of Ru enhances the Ni reducibility and leads to greater Ni dispersion on the catalyst surface, thus promoting overall activity and CO selectivity for the RWGSR (Sache et al., 2020). Typically, MOF-74 plays a role in helping adsorb and deliver electrons, whereas the low amount of Au@Pd NPs in Au@Pd@MOF-74 results in the poor photon adsorption strength of the Au@Pd active sites. Based on this feature, the core-shell Au@Pd@MOF-74 nanostructure is more propitious to generate CO than MOF-74 in the RWGSR because CO generation is a two-electron reaction, while CH_4_ generation requires eight electrons; thus, it is more difficult to produce CH_4_ (Han et al., 2019). Because of the functional characteristics of Au@Pd nanoparticles, the Pt/Au@Pd@UIO-66 catalyst is synthesized to improve its catalytic activity in the RWGSR, as shown in Figure 6. In this system, the core-shell monodispersed Au@Pd nanosphere is encapsulated in the UIO-66 to control its morphology and impart nanoparticle functionality. Additionally, the microporous nature of the UIO-66 assists the adsorption of the Pt nanoparticles, which enhances the interaction between them, favoring the formation of isolated and well-dispersed Pt nanoparticle active sites. This advanced architecture results in excellent catalytic activity and CO selectivity for the RWGSR, and the concept of inserting nanoparticles into microporous MOFs will revolutionize future industrial applications (Zheng et al., 2018). DFT calculations indicate that the catalytic behavior of a Cu_12_TM (TM = Co, Rh, Ir, Ni, Pd, Ag, Au) bimetallic nanocluster in the RWGSR is dependent on the position of the d-band center. In general, the closer the d-band center is to the Fermi level of these catalysts, the greater is the CO_2_ adsorption energy, and the smaller is the C-O bond dissociation barrier. Therefore, Cu_12_Co delivers better catalytic activity for the RWGSR, with a TOF value of 8.96^*^10^−13^ s^−1^, than do the Cu_13_ and Cu_12_TM bimetallic systems, due to its d-center value of −0.547 eV, which is higher than that of the other two systems (Zhang and Guo, 2018). Furthermore, for γ-Al_2_O_3_- and CeO_2_-supported Co catalysts, with the addition of Pt as the second component, the values of the d-band center move from the Fermi level toward more negative values, which prevents the excessive hydrogenation of the C-O bond of the CO_2_ molecule and thereby increases the CO selectivity of the RWGSR (Porosoff and Chen, 2013). In addition, the deposition of Mo onto Au/SiO_2_ catalyst generates new Au/MoO_x_ interfacial sites since it preferentially occurs on undercoordinated Au sites. The heat of CO adsorption (ΔH_ads_) for the Au/MoO_x_ sites is 33 kJ mol^−1^, considerably lower than that of the Au^0^ sites, indicating that these interfacial sites are more selective than the Au^0^ sites for the RWGSR (Carrasquillo-Flores et al., 2015). Similarly, the Cu/Fe interfacial active sites are generated after introduction of additional Fe into the Cu/SiO_2_ catalyst, on which the formation of the Fe-Cu bond also prevents Cu from being sintered and oxidized during the RWGSR (Chen et al., 2004). Moreover, the addition of Cu to the Mo/FAU catalyst results in an improvement in the reducibility of MoO_3_. Therefore, the Mo(0.8)Cu(0.2)/FAU catalyst, which contains co-supported Mo-Cu at an atomic ratio of 4:1, exhibits the higher CO yield of 18.5% and selectivity of 99% compared with the supported Mo catalyst for the RWGSR with the feed gas (H_2_:CO_2_ = 1:1) at atmosphere pressure and 773K (Okemoto et al., 2020). The bulk Pt_3_Ni intermetallic parent compound is formed selectively over the Pt-Ni bimetallic catalysts supported on mesoporous silica, which is related to the thermodynamics of the phase equilibria with a metal silicate that precludes the formation of more Ni-rich intermetallics during the operando conditions of the RWGSR, as shown in Figure 7. This proposed intermetallic structure for these ~1 nm supported clusters, shows a surface/interfacial speciation of the Ni in which only heterometallic Pt-Ni interactions are present in an atomic arrangement within the catalytically active bimetallic sites, which afford exceptionally high activity and CO selectivity in the RWGSR (Liu et al., 2018).

#### Alkali Promoter Effect

For supported heterogeneous catalysts, alkali metal components are typically introduced as promoters to increase the amount of adsorption sites and mediate the adsorption strength of the reactants and intermediates on the “inert support” (Li et al., 1998; Gálvez et al., 2013; Obalová et al., 2013; Connor and Holland, 2017; Pacultová et al., 2017). For instance, the addition of alkali metal promoters is crucial for industrial catalysts substantially applied in the Fisher-Tropsch synthesis (Mirzaei et al., 2006; Okabe et al., 2007; Feyzi et al., 2011; Cosultchi et al., 2012) and ammonia synthesis (Shimoda et al., 2017; Lin et al., 2018a; Jafari et al., 2019; Rogowski, 2019; Zhou et al., 2019). In the field of RWGSRs, abundant studies have shown that K and other promoter additives are essential for some supported catalysts to acquire the expected CO product (Arunajatesan et al., 2007).

**Figure 8:**
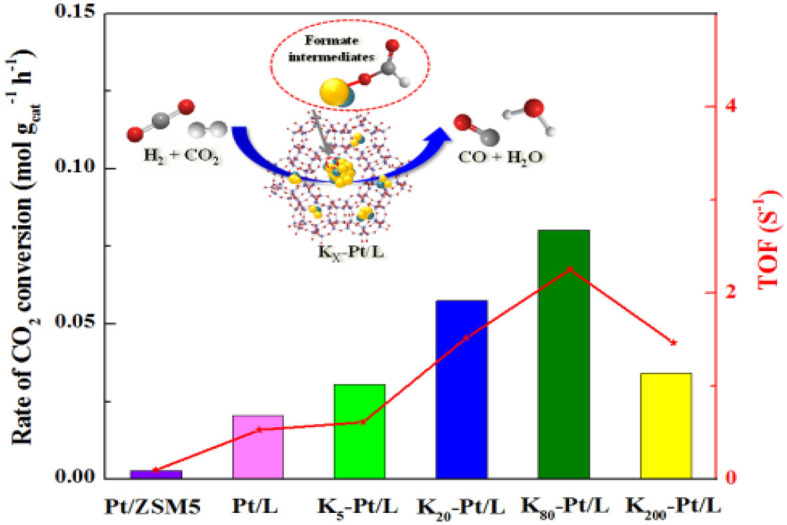
RWGSR over Pt/L catalysts with different K contents. Reprinted with permission from Yang et al. (2017). Copyright (2017) Elsevier.

The highly K-promoted Fe/Al_2_O_3_ and Cu/SiO_2_ catalysts give much higher CO formation rates than do their counterparts in the RWGSR mainly because the addition of K introduces abundant weak, medium, and strong basic sites, which helps to adsorb/activate CO_2_ and further converts the CO_2_ to CO through reaction (Choi et al., 1996; Chen et al., 2003). For the Ni/Al_2_O_3_ catalyst, carbonates are the main intermediates, decomposition of which to CO is relatively difficult. However, *via* modification of this catalyst with a strong base such KOH, the formates rather than the carbonate forms as the main intermediate, which strongly absorbs on the surface of the Ni-KOH/Al_2_O_3_ catalyst, contributes to CO formation and hinders further hydrogenation of the CO to CH_4_ through C-O bond scission (Zhang et al., 2019a). Furthermore, it is demonstrated that the introduction of alkali promoter (K, Mg) by co-impregnation technique enhance the dispersity of Ni active species on the Al_2_O_3_, thus increasing the RWGSR performance (Ranjabar et al., 2019). The addition of K promoter leads to an electron transfer from Pt to O in KO_x_ species, resulting in the generation of interfacial active sites over the Pt/mutille catalyst, which is proposed to be more responsible for the production of CO (Liang et al., 2017). Similarly, the K promoter acts as a reducing agent relative to the Fe metal, and the observed increase in the ratio of the Fe^2+^/Fe^3+^ ions over the BaFe-hexaaluminates after the K addition reflects the increasing concentration of reduced Fe^2+^ ions in the hexaaluminate lattice, which is accompanied by the appearance of oxygen vacancies due to the cleavage of one of the neighboring Fe-O-M (M = Al, Ba) bonds in the first coordination sphere of Fe ions. These vacancies play a role in the sites for CO_2_ adsorption forming monodentate surface carbonates followed by redox transformation evolving CO and leaving the second oxygen bonded to the Fe^3+^ ion (Wang et al., 2013b,c; Utsis et al., 2018). The results of DFT calculations demonstrate that the K adatom greatly stabilizes the adsorption of all oxygenate intermediates through direct K-O bonding formation on K-modified Cu(111) and Cu(110) surfaces, thus promoting CO_2_ dissociation in the RWGSR. In general, the different promoting effects of alkali metals on CO_2_ dissociation are due to their electronegativities, which induce different work function changes and surface dipole moments. Correspondingly, the promoting effects on CO_2_ dissociation induced by alkali metals increase in the order of Na < K < Rb < Cs, while the electronegativity of various alkali metals decreases in the order of Na > K > Rb > Cs (Wang and Wang, 2019). In accordance with the above effects, the electronegative character facilitates the electronic transfer from Cs to Mo and Fe and leads to an electronically rich surface, which favors the selectivity toward CO over the corresponding catalysts (Pastor-Pérez et al., 2018; Zhang et al., 2019b). For the WC/γ-Al_2_O_3_ catalyst, the addition of the K promoter not only has a structural effect to promote the dispersion of the WC species across the high surface area support but also can serve as electronic promotion to strengthen CO and CO_2_ adsorption while weakening H_2_ adsorption, which is therefore hypothesized to result in a lower H_2_/Co_x_ on the catalyst surface, thus inhibiting hydrogenation activity for CH_4_ and accelerating the generation of CO *via* the RWGSR (Morse et al., 2020). Specifically, our studies have systematically investigated the effect of K promoter on the activities and selectivities of zeolite L-supported Pt catalysts for the RWGSR, as shown in Figure 8. This study concluded that an additive K promoter not only alters the work function of Pt through their interaction but also forms Pt-O(OH)-K interfacial sites. In addition, the electronic properties of Pt-O(OH)-K sites, with a charge transfer from the Pt surface to the adjacent O in KO_x_, facilitate the formation of formate intermediates and desorption of the CO. However, with excessive addition of K, the access of the reactants to the Pt surface and interface is tightly blocked. Thus, the activity of the RWGSR is significantly promoted by the controlled addition of K promoter (Yang et al., 2017).

#### Oxide Catalysts

Reducible transition oxides are intensively employed in RWGSR catalysts due to their relative abundance and their OSC, which is the ability to reversibly store and release oxygen while formally switching the valence state of the metal ion in its own structure under a CO_2_/H_2_ atmosphere (Reddy et al., 2010; Dong et al., 2012; Yao et al., 2013). The O atom can be deprived of H_2_ in the oxide lattice to generate surface oxygen vacancies, which are much more favorable to the generation of CO rather than CH_4_, because the oxygen from the C-O bond cleavage of the CO_2_ molecule can be accommodated, but this leads to unsatisfactory catalytic activity and thermal stability (Katta et al., 2010; Ahn et al., 2012; Graciani et al., 2014). Normally, the additional introduction of heteroatoms into the oxides leads to the formation of spinel, solid solution, and perovskite-type oxides, and their ultra-stable structure is conductive to reversible oxygen donor-acceptor over oxygen vacancies sites, which thus effectively overcome the disadvantages of pure oxides as RWGSR catalysts (Ringuedẽ and Fouletier, 2001; Royer et al., 2005).

ZnO-based oxides are preferentially utilized to catalyze the RWGSR during the CAMARE process due to its ease of formation of metal composite oxides with high stability and specific activity (Li et al., 2002; Schmale et al., 2013). Nonetheless, ZnO-based oxide catalysts are vulnerable to the reduction of ZnO to Zn metal and therefore the loss of ZnO active components when exposed to high thermal reaction conditions, which contributes to their catalytic deactivation (Park et al., 2001). The formation of the spinel structure of the ZnAl_2_O_4_ phase by addition of Al_2_O_3_ to the ZnO catalyst can cause resistance to its catalytic deactivation in the RWGSR (Joo and Jung, 2003). Similarly, when Fe_2_O_3_ is substituted by ZnO over the Fe_2_O_3_/Cr_2_O_3_ catalyst, the corresponding ZnCr_2_O_4_ phase is formed and thus becomes stable (Park et al., 2000). In the synthetic process of the ZnZrO_x_ mixed oxide, the substitution of the Zr in the first layers of the *m*-ZrO_2_ lattice with Zn causes the formation of a surface solution (Zn_x_Zr_1−x_O_2−y_), which generates oxygen vacancies and improves its stability, reducibility, and oxygen mobility, thus increasing the CO_2_ conversion in the RWGSR (Silva-Calpa et al., 2016).

The widespread application of CeO_2_-based oxide in RWGSR catalysts is mainly due to its high OSC, which is inextricably correlated with the catalytic activity (Masui et al., 1997; Wang and Liu, 2018). Both the manipulation of the CeO_2_ shape with emphasis on tuning its fraction of reactive crystal planes and the doping CeO_2_ with heterocations to alter its structure and chemical properties are effective strategies to obtain a superior OSC (Sun et al., 2012; Zhou and Li, 2012). Considering their distinct morphologies (particles, rods, and cubes), the higher activity of CeO_2_ cubes in the RWGSR is due to the superior inherent reactivity of the CeO_2_ (100) crystal planes enclosing the cubes, contrary to the less inherently reactive CeO_2_ (111) facets enclosing the rods and particles in the RWGSR (Kovacevic et al., 2016). The CeO_2_ lattice distortion caused by the incorporation of heterocations such as Zn increases the oxygen vacancy defects and thus accelerates the mobility of the oxygen ions, leading to a higher OSC, thus markedly enhancing its catalytic activity in the RWGSR (Lin et al., 2015; Wenzel et al., 2017). In addition, either Ce_0.75_Zr_0.25_O_2_ or Ce_0.75_Zr_0.5_O_2_ solid solution can be formed by the addition of Zr to the CeO_2_ lattice, increasing its ability to generate oxygen vacancies and, more importantly, promoting its thermal stability, which is a very promising aspect of catalytic systems employed in reactions in which the RWGSR is one of the steps in the processes that generate hydrocarbons from CO_2_ (Zonetti et al., 2014; Wenzel et al., 2018).

**Figure 9:**
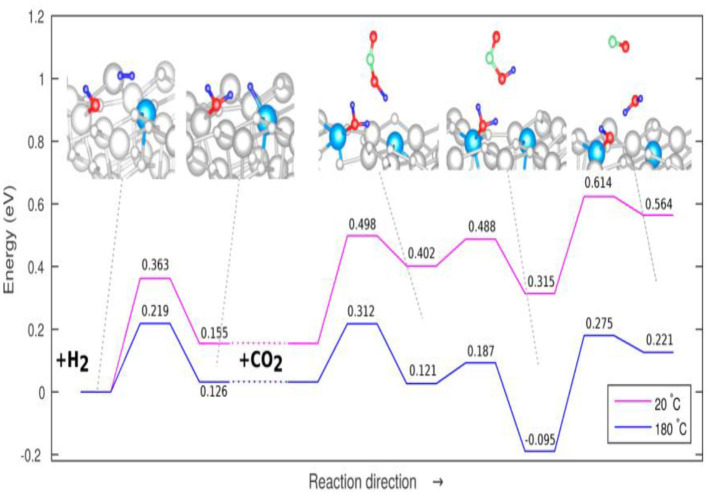
Overall proposed mechanism for the RWGSR on In_2_O_3−x_(OH)_y_ at 20°C (pink line) and 180°C (blue line). Reprinted with permission from Ghoussoub et al. (2016). Copyright (2016) American Chemical Society.

The adsorption of CO_2_ on In_2_O_3_ has an adsorption energy of −1.25 eV, which is sufficiently exothermic and thus favorable, so the O-C-O angle of the CO_2_ on adsorbed on the In_2_O_3_ is significantly distorted relative to the gas phase structure, significantly increasing the activity of the CO_2_ in the RWGSR (Ye et al., 2012; Sun et al., 2014). The oxygen vacancies are increasingly created and stabilized on In_2_O_3_ with the presence of CeO_2_ in the In_2_O_3_-CeO_2_ catalyst, on which the dissociated H_2_ adsorption is enhanced and the amount of bicarbonate species resulting from activated CO_2_ is increased, which thus exhibits enhanced catalytic activity for the RWGSR (Wang et al., 2016c). Cubic In_2_O_3_[denoted as c-In_2_O_3_(110)] exhibits a higher RWGSR rate than the hexagonal In_2_O_3_[denoted as h-In_2_O_3_(110)] at temperature below 350°C due to its enhanced dissociative adsorption of H_2_, facile formation of the oxygen vacancies, and enhanced ability to adsorb and activate CO_2_ on the oxygen vacancies (Wang et al., 2020). DFT calculations indicate that the oxygen vacancies sites on the In_2_O_3_ (110) surface assist CO_2_ activation and hydrogenation and stabilize the key intermediates involved in CO formation (Ye et al., 2013). Furthermore, an In_2_O_3−x_(OH)_y_ surface containing both Lewis base hydroxide groups and Lewis acid In sites together with oxygen vacancies can heterolytically dissociate H_2_ to form a hydride bonded to In metal and a proton bonded to a lattice O. This hydrogenated In_2_O_3−x_(OH)_y_ surface facilitates CO_2_ reduction by mediating the charge transfer between the In_2_O_3−x_(OH)_y_ surface and adsorbed reactants CO_2_ and H_2_ to form CO and H_2_O (Ghuman et al., 2015). Well-tempered MetaD-biased AIMD simulations have been performed, taking the temperature into account, to probe the mechanism for the RGWS reaction over the In_2_O_3−x_(OH)_y_ surface at temperatures of 20 and 180°C, as shown in Figure 9, and the results show that the reduction of gaseous CO_2_ is the rate-limiting step, with no significant change resulting from increased temperature. However, the energy barrier corresponding to the adsorption of CO_2_ is slightly reduced at 180°C compared to the that at 20°C, suggesting that the thermal effects may only be relevant to the reaction step characterized by an adsorptive mechanisms and that the increased thermal conditions may enhance the reactivity by enabling the surface frustrated Lewis pairs to become further spatially separated (Ghoussoub et al., 2016).

**Figure 10:**
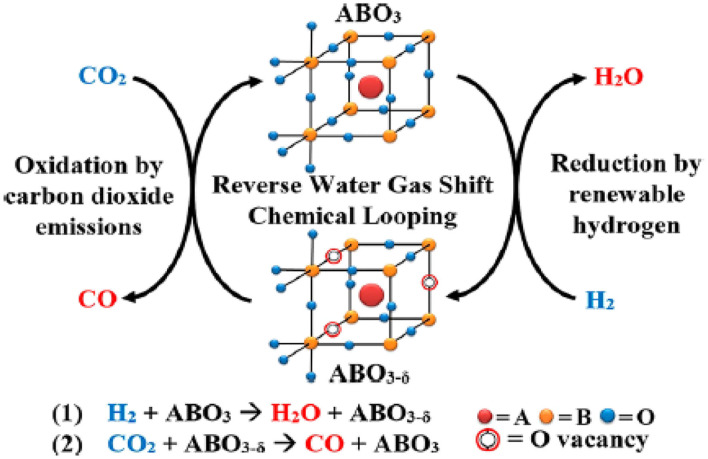
Reverse water gas shift–chemical looping process. Reprinted with permission from Ringuedẽ and Fouletier (2001). Copyright (2018) American Chemical Society.

The perovskite-type oxides are represented by an ABO_3_ formula, where the A-site is typically occupied by lanthanides or alkaline earth metals, and the B-site is usually filled with transition metals (Yamazoe et al., 1981; Peña and Fierro, 2001; de Lima et al., 2009). With multiple cation combinations possible on each site, perovskite-type oxides can be easily customized to achieve desirable properties, such as high oxygen mobility and tunability, together with thermal stability at high temperatures without aggregation (Royer et al., 2005; Kae et al., 2014). Therefore, these materials are attractive for application to the RWGSR with chemical looping cycles (RWGSR-CL) that can convert CO_2_ and H_2_ to separate streams of CO and H_2_O, as depicted in Figure 10 (Ringuedẽ and Fouletier, 2001). The combination of La and Sr in the A-site and metal in the B-site enhances the formation of oxygen vacancies due to the generation of a charge imbalance in the ABO_3_ structure caused by the difference in their oxidation states (Daza et al., 2014). Regarding Co-based perovskite type oxides (La_0.75_Sr_0.25_CoO_3−δ_), under H_2_ flow conditions, their phases can change to metallic cobalt and base oxides (Co/SrCO_3_/La_2_O_3_), which are then reoxidized to a layered perovskite (CoO/LaSrCoO_4−δ_) with a K_2_NiF_4_-type structure when exposed to CO_2_, thus producing CO during this cycle. Additionally, the optimal isothermal reduction and conversion temperatures for maximizing the CO product rates of 113.9 μmole CO/g/min are 500°C (of 400, 500, and 600°C) and 850°C (of 650, 750, and 850°C), presumably due to the formation of mixed oxides and metallic cobalt crystalline phases (observed via X-ray diffraction) in close contact under these conditions (Royer et al., 2005). Fe-based perovskite type oxides [La_0.75_Sr_0.25_FeO_3_ (LSF)] have shown the greatest promise in the RWGSR-CL process due to the low energy barrier for oxidation-state transitions (Fe^3+^-Fe^2+^) during the redox cycles (Peña and Fierro, 2001). Enhanced oxygen self-diffusion, material recyclability, and therefore the viability of LSF have been demonstrated for chemical looping when supported by redox materials with more abundant alternatives, such as CeO_2_, ZrO_2_, Al_2_O_3_, SiO_2_, and TiO_2_ (Li et al., 2011; Chen et al., 2014). In comparison, supports such as TiO_2_ and Al_2_O_3_ demonstrate SMSIs, which often result in some degree of LSF particle encapsulation, even at low temperatures, thus hindering the CO_2_ adsorption on the surface oxygen vacancies, whereas SiO_2_ demonstrates more moderate interactions that are strong enough and suitable for particle segregation yet weak enough to avoid deactivation (Min et al., 2003; Hare et al., 2019). These behaviors occur because the utilization of SiO_2_ as a support significantly reduces the average LSF crystallite size and the extent of oxygen self-diffusion retardation, and the CO generation yields of LSF/SiO_2_ surpass those of LSF alone by ~200%, producing 2.6 mmol of ${{\text{CO}}_{\text{gLSF}}}^{-1}$ at a peak rate of 0.8 mmol ${{\text{CO}}_{\text{gLSF}}}^{-1}$ min^−1^ (Hare et al., 2018). In addition, further modification of Fe-based perovskite type oxides with transition metals helps to increase the strength of the interaction of the active species and support and thus stabilizes the unusual cationic oxidation state in the RWGSR process (Nitarori et al., 1988). The incorporation of Cu in La_0.75_Sr_0.25_Fe_1−Y_Cu_Y_O_3_ perovskites [Cu100^*^Y (with Y = 0, 0.10, 0.25, 0.50, 0.75, and 1)] facilitates the formation of oxygen vacancies at lower temperatures. CO production is promoted in the Cu10 sample vs. Cu0 and Cu25, likely due to a combined effect of better CO_2_ dissociative chemisorption energies on metallic Cu and decreased thermodynamic stability of the oxygen-deficient perovskites (Daza et al., 2016). The enhanced crystalline structure stability is aroused by the incorporation of Co in the La_0.75_Sr_0.25_Co_(1−Y)_ Fe_Y_O_3_ perovskite. Additionally, a computational investigation using DFT calculations correlates CO_2_ adsorption strength, generally a strong barrier in CO_2_ conversion, on the (100) crystal facets on La_0.75_Sr_0.25_FeO_(3−δ)_ to increasing the surface oxygen vacancies (δ). Therefore, δ in the perovskite is the driving force to break the CO-O bond and reoxidize the La_0.75_Sr_0.25_FeO_3−δ_ (Daza et al., 2015; Maiti et al., 2016).

## Conclusion and Outlook

The large-scale conversion of CO_2_ to CO *via* the RWGSR is a promising route with great potential for use in the near future, provided a mature technology for commercial production of renewable H_2_ is also available. The RWGSR also achieves higher CO_2_ conversion than other relevant technologies that meet the global CO_2_ emissions standards. Because it is a slightly endothermic and pressure-independent reaction, the current challenge for RWGSR employed in fuel synthesis is the design of thermally stable materials that can achieve high CO selectivity and high production rates. Preferential strategies have recently been enacted to address the existing problems either by modulating the SMSI, size of the active metal, second metal composition, and addition of alkali promoter for supported catalysts or by dipping with additional heteroatoms or tuning their crystal planes for oxide catalysts. Furthermore, the perovskite-type oxides can act as the oxygen donor-acceptor for the RWGSR-CL to not only circumvent thermodynamic and kinetic limitations but also eliminate the possibility of methanation as a side reaction because there is no direct interaction between two feed gases and between two product streams.

From this systematic introduction, the relationships between the nature of the active sites and the main intermediates of RWGSR catalysts are understood through the insights gained from the molecular dynamic simulations and mechanistic work under the operando reaction conditions, which is beneficial to the development of state-of-the-art architecture of RWGSR catalysts. However, even though several materials have been studied, improvements are still possible, especially for commercial development of RWGSR catalysts for laboratory and market applications. If the RWGSR plays a major role in the reduction of the atmospheric CO_2_ concentration, then designing catalysts with earth-abundant materials will be necessary and desirable. To develop supported metals, both Fe oxides and Ni oxides are chosen to be investigated as representative substitutes for the most commonly used reducible supports, such as CeO_2_, ZnO, and In_2_O_3_, largely due to their oxygen vacancies with high oxygen mobility and stability, which can activate CO_2_ more easily by accommodating oxygen due to the C-O bond cleavage in the RWGSR. Additionally, transition metal carbides are attractive and convenient alternatives for industrial use in the RWGSR because of their properties, which are similar to those of precious metals, as well as their dual functionality for H_2_ dissociation and C-O bond scission and their potential to behave similarly to reducible oxides.

## Author Contributions

XC, CS, and YC drafted the manuscript, conceived the concept of the review, conducted literature survey, and arranged the figures. NW provided the suggestions. WW and LC revised the manuscript and provided comments. All authors contributed to the article and approved the submitted version.

## Conflict of Interest

The authors declare that the research was conducted in the absence of any commercial or financial relationships that could be construed as a potential conflict of interest.
